# The Progress and Policy of the Hospitals Association

**Published:** 1893-01-28

**Authors:** 


### The Hospital

An Institutional Journal of

Science, Medicine, Nursing, and Philanthropy.

*For Hospitals, Asylums, Infirmaries, Dispensaries, Medical Practitioners, Students, Nurses, and the Charitable Public.*

Vol. XIII.—No. 331.] [Jan. 28, 1893.

### The Progress and Policy of The Hospitals Association.

The Hospitals Association was founded in 1884, and inaugurated on the first of February at a meeting held at the Mansion House under the presidency of the Lord Mayor. At the outset it was created as a working substitute for a Royal Commission, which progressive hospital workers had long asked for in vain. It was established with the object of bringing order out of chaos. Too much individualism, like too many cooks with the broth, was threatening to "spoil" the hospitals. The Association has had a singularly eventful, useful, and successful history, and it may be well to take a brief retrospect of its aims and its past work before indicating what the lines of its future policy might wisely be.

The Hospitals Association aimed at bringing together for conference and mutual help accredited representatives of the voluntary hospitals both from London and the country. In this it succeeded excellently well. It is true that in a little time some of the less patient and less disciplined spirits "jumped away" at one or more tangents. That was inevitable. There were "separatists" in things great and small long before the Irish question threatened to dismember the Empire; and persons of strong impulse but feeble judgment will recruit the ranks of separatism to the end of time. But the original kernel of loyalty to reasonable methods and progressive aims never deserted the flag. True men did true service, and gradually brought the Association from victory to victory, until even its enemies were reduced to the melancholy expedient of snarling at successes which they did not fail to envy, but which they never so much as even attempted to imitate.

Directly or indirectly, The Hospitals Association has been the originator of The Hospital newspaper, the Royal National Pension Fund for Nurses, the British Nurses' Association, the Metropolitan Service of Street Ambulances, and of all the most impo:tant hospital liteiature which has been produced during the last ten years. This is a pretty considerable record for nine years' work, but it might be added to indefinitely. Through the instrumentality of the Association hospitals have been freed from the numbing paralysis of the statute of mortmain; they have been vindicated from the charge of sectarianism; their splendid services have been brought prominently into public recognition; their methods have steadily improved; their value has been more and more clearly perceived; the Press and the public have moderated the acridity of their criticism, and, we venture to say that, at the present time, thanks very largely to the persistent patience and reasonableness of The Hospitals Association, the hospitals of this country occupy an exalted position in the public regard such as they never occupied before.

The Hospitals Association has good reason to be proud of most of its offspring. Even the British Nurses' Association is not without merits. It is good that nurses should gather themselves together for mutual counsel and help. If the leaders of the B.N.A. were well advised they would be more severely practical in their aims, and less obtrusively flamboyant in their methods. It is a serious mistake, too, for so considerable a minority of nurses to tear themselves so violently and even passionately away from the hospitals and nurse training schools. Unhappily, the B.N.A. is largely controlled by persons inexperienced in affairs, and it must inevitably, therefore, comport itself after the manner of inexperience. But such as it is, it owes its origin to The Hospitals Association.

It hardly becomes the present writer to say anything in praise of The Hospital. Its circulation and its advertisement pages tell their own story to those who can read. Our point at present is that The Hospital is one of the many stalwart and successful children of the The Hospitals Association. The Royal National Pension Fund may be more freely spoken about. The writer has no official connection with it; though from the first he has had full knowledge of its working, its struggles, and its successes. The Pension Fund is a splendid achievement. To-day its invested funds amount to no less a sum than £135,000, of which more than £80,000 consist of nurses' contributions alone. During the past six months nurses have been joining the Fund at the rate of forty-seven per month, and during the last three months at the rate of a little more than fifty-six per month. If this is not permanent, striking and most gratifying success, perhaps critics and petulant enviers will tell us what would be?

Of the Street Ambulance Service which the Association has created and so efficiently organised, what is it necessary to say? Positively nothing! Everybody knows that the sum of £1,500 has been expended in Hand Ambulances, and that those ambulances are now so judiciously distributed over the greater part of London, and so competently manned, that a dangerous accident in the smallest street commands attention at once, and the suffering victims are transferred to the nearest hospital without so much as a moment's unnecessary delay. This is an invaluable service to have rendered to the millions of frequenters of the London streets.

Space forbids us to speak at any length of the large mass of hospital literature which owes its birth to The Hospitals Association. That literature is of the most practical and necessary kind. Finance, nursing, general administration, apologetics, hospital sociology and a hundred other departments of hospital activity have been dealt with in turn by experienced and competent writers. All this mass of literature remains as a permanent monument of the work of the Association; and not as a monument only, but as a mine of literary wealth from which future hospital workers may dig and constantly enrich themselves, and enhance the value of their work. To take as a solitary example the book published under the title of "Suffering London." The book was written by Mr. Egmont Hake, and introduced by Mr. Walter Besant in a valuable and characteristic preface. It was warmly welcomed by the general and medical press, and up to the present time, within a year of its first issue, no fewer than 2,500copie s have been asked for. This is a remarkable success. The book has done, and will continue to do, its work. We have reason to believe that Baron Hirsch is a reader of The Hospital and of works like "Suffering London"; and it is probably largely due to the influence of such reading that he has recently sent such magnificent contributions to so many struggling hospitals. That Baron Hirsch's noble example will prove universally contagious we need not doubt.

What, then, is to be the future policy of this unassuming but useful Association? Its immediate policy must be one of expectancy. It will welcome the formation of a representative Central Board for London on the lines proposed by Lord Sandhurst, and if such a committee is ultimately constituted it will have no more loyal supporters than the present members of The Hospitals Association. But will such a Central Board be formed? The meeting at the School Board offices on Tuesday seems to have answered this question by anticipation, and answered it in the negative. The meaning of this is that the Lords' Committee, which took so much pains, both to elucidate the facts and to reach sound conclusions, is to be absolutely barren of all practical results. If this should prove to be indeed so, it will be nothing short of a public scandal. In the meantime The Hospitals Association, instead of abdicating in favour of a wider and more truly representative organisation, must not only continue to hold its place but to work at the various problems of hospital economy in a resolute and progressive spirit. But if contrary to present appearances the day should come that opposed parties lay down their weapons of opposition, and by mutual concessions join hands in the great work of statesmanlike hospital legislation, the Association will be the first to help forward the newer life and work; until that day dawns it will, as we have said, maintain the ground it has won, and hopefully strive to conquer more.

